# Evaluating interprofessional primary care teams in high-income countries: A scoping review protocol on the conceptualization and measurement of team functioning, effectiveness, performance and collaboration in primary care

**DOI:** 10.1371/journal.pone.0328708

**Published:** 2025-07-18

**Authors:** Monica Aggarwal, Ivy Bourgeault, Sara Dalo, Kristina M. Kokorelias, Leslie Greenberg, Bill Kreutzweiser, Kevin Samson, Leslie Sorensen, Connor Kemp, David Schieck, Ross E. G. Upshur

**Affiliations:** 1 Dalla Lana School of Public Health, University of Toronto, Toronto, Ontario, Canada; 2 Department of Family and Community Medicine, University of Toronto, Ontario, Canada; 3 School of Sociological and Anthropological Studies, University of Ottawa, Ottawa, Ontario, Canada; 4 Tilbury District Family Health Team, Tilbury, Ontario, Canada; 5 Section of Geriatrics, Department of Medicine, Sinai Health and University Health Network, Toronto, Ontario, Canada; 6 Department of Occupational Sciences and Occupational Therapy, University of Toronto, Toronto, Ontario, Canada; 7 Rehabilitation Sciences Institute, University of Toronto, Toronto, Ontario, Canada; 8 Ontario College of Family Physicians, Toronto, Ontario, Canada; 9 Section on General and Family Practice, Toronto, Ontario, Canada; 10 Association of Family Health Teams of Ontario, Toronto, Ontario, Canada; Lahore Medical and Dental College, PAKISTAN

## Abstract

**Introduction:**

The delivery of primary care (PC) services by interprofessional teams serves as the cornerstone for building high-performing PC systems. Interprofessional team-based care is a collaborative approach to primary care delivery where healthcare professionals from multiple disciplines work together to provide comprehensive and coordinated care. Despite this recognition, the assessment of the impact of interprofessional PC teams is limited or mixed. There is a lack of clarity on how to define and measure team functioning, collaboration, performance, and effectiveness in PC, posing challenges for the evaluation of interprofessional PC teams. This review aims to dissect and analyze the definitions (conceptualizations and operationalization), measures, and measurement methodologies employed in defining and evaluating team functioning, collaboration, performance, and team effectiveness in PC. In the context of interprofessional PC teams, this review will answer the following questions: 1) How are the terms team functioning, performance, effectiveness, and collaboration conceptualized? 2) What are measures of team functioning, performance, effectiveness, and collaboration? 3) What instruments are used to evaluate team functioning, performance, effectiveness, and collaboration?.

**Methods:**

A systematic approach will be undertaken to conduct this review. A comprehensive search across various academic databases, including PubMed, Medline, CINHAL, Scopus, and Web of Science, will be conducted. Keywords such as “team functioning,” “performance measurement,” “team effectiveness,” “team collaboration,” “primary care,” and “primary healthcare” will be utilized to ensure the inclusion of relevant studies. Inclusion criteria will be established to filter studies focusing explicitly on interprofessional teams. The review will encompass both qualitative and quantitative studies, ensuring a holistic understanding of the subject matter. By synthesizing this information, the review aims to present an encompassing overview of the conceptualization, measurement and instruments employed to evaluate team functioning, performance, effectiveness, and collaboration within PC settings.

**Discussion:**

Globally, governments are investing in the implementation of interprofessional PC teams. The lack of clear definitions and measurement of team outcomes underscores the importance of conducting a comprehensive review. This review will aim to address this gap in knowledge and help inform practice and policy, ultimately contributing to optimizing team functioning, performance, effectiveness, and collaboration within PC settings.

## Introduction

Primary care (PC) is the cornerstone of healthcare systems [1]. PC is often the initial point of contact for individuals seeking medical care and provides a wide range of preventive care, disease management, and coordinated services tailored to individual and community needs [2]. The World Health Organization defines primary health care (PHC) as the integration of PC with public health, premised on community participation and multisectoral policy and action [3]. The goal of primary care reform is increasingly to shift towards population-focused and community-oriented approaches to address the social determinants of health. As such, we define PC “broadly to cover the spectrum of first-contact health care models that focus on comprehensive, person-centered care sustained over time along with PHC initiatives that incorporate health promotion, community development, and addressing the social determinants of health” [4] pg.7 [1] p. 1140–1141.

Over decades, primary care reform initiatives have focused on shifting from traditional solo physician-led practice delivery models to interprofessional team-based models in PC across global jurisdictions [5–8]. Interprofessional teams are seen as the solution to responding to the challenges of the healthcare system and are expected to improve patient attachment to PC professionals [9,10] and improve workforce retention and job satisfaction [11–13]. Interprofessional team-based care refers to a model of care where healthcare professionals from various PC disciplines (e.g., physicians, nurses, pharmacists, social workers and others) work together to deliver coordinated services to meet the various healthcare needs of patients and communities [14,15]. Although there are studies that show interprofessional teams are beneficial to the care of patients with chronic and medically complex conditions [16–19], there are several studies that show limited or conflicting evidence of impact [14,20–24]. This is in part due to ‘teams’ not being a unitary nor standardized concept.

Interprofessional PC teams are complex and varied. At the organizational level, there is significant variability in the characteristics in which interprofessional teams work together. This includes how the organization is governed, how the different team members are paid and how the team is organized. The infrastructure of the PC team may operate under one community, provider, or mixed governance model or as two separate entities – one for physicians and the other for interprofessional PC professionals [25–27]. Requirements for performance measurement and accountability can also differ across models [28–31]. The team can be globally funded with direct payment arrangements with PC professionals, or physicians can be paid directly as independent contractors through different remuneration models [14]. There can be differences in the basket of services delivered to the patient population based on the funding model and team composition [32]. Service requirements can vary, with some organizations requiring patient enrolment, delivery of after-hours care, etc. [33].

At the interprofessional team level, a group(s) of PC professionals can be led by one or more leaders with a focus on clinical and organizational roles [34]. The size and composition of the interprofessional team can include a few or several PC professionals both within and across cadres [35,36]. Interprofessional PC professionals can be employees of the team or serve as a resource to the team through a referral-based model. Different PC professionals have both distinct and sometimes complementary or overlapping scopes of practice, resulting in differences in roles, responsibilities, and authority within the team. These roles, autonomy and scopes of practice can be implemented differently based on team composition, practitioner preference or organizational culture [7,8,15,37–40]. PC professionals may work in the same physical location or work virtually across various practice sites in the community [41]. There might be processes in place to enable team communication (electronic records, meetings, protocols), or care may be delivered in silos. The size and nature of the population the team serves can also be different, with some models focused on providing care to the general population while others working with specific populations (i.e., rural or marginalized populations) [15].

A significant challenge for interprofessional PC professionals is joining a team without the prerequisite knowledge and skills to collaborate with a diversity of PC professionals. Existing literature demonstrates that innovative PC practices include various healthcare professionals and lay staff that work to their full potential to meet the needs of their patient population [7,15,42]. However, structural (characteristics of the workplace), interpersonal (dynamics between team members such as trust and leadership) and individual dynamics (personal attributes) have an important role in defining role boundaries and distribution of tasks [38,43,44]. Hierarchical dynamics between physicians and non-physicians, in particular, have been shown to impede effective collaboration and communication among team members, leading to disparities in decision-making authority and provision of services [45–48]. These challenges may explain the limited or mixed results of the impact of interprofessional PC teams, highlighting the pivotal need to understand how teams can work more effectively together and achieve the goals of the quintuple aim. Improving team functionality necessitates first defining the characteristics of high-functioning teams, which include effective communication, trust, collaboration, strong leadership, and adaptability [42].

Through a previous scoping review on PC teams, we discovered that foundational knowledge of the conceptualizations and measurements of team outcomes [14] is lacking. In the literature, the terms related to team performance and team process are often not defined or used interchangeably. The terms “teamwork” and “team functioning” are used interchangeably, which may be inappropriate [49]. These terms are defined to include collaboration [50,51], performance [52], and effectiveness [53,54]. Team functioning is operationalized to include conflict resolution strategies and adaptability, which may be distinct from teamwork [49]. Collaboration is defined as the cooperative efforts of team members to achieve common goals [55] and having a team vision [56–59]. The term “collaboration” has also been intertwined with “team functioning” [60] and “team effectiveness.” In some cases, team collaboration has been identified as an outcome of team functioning [61,62], which, in turn, enhances team performance and effectiveness in delivering care [62–64]. In other cases, team collaboration and performance contribute to team functioning and effectiveness. Through a comprehensive examination of the literature, a scoping review can help identify the different conceptualizations, frameworks, and operationalizations of these terms, providing a clearer understanding of their meanings and how they are used in research and practice contexts.

The lack of clear definitions of these terms poses a significant challenge in understanding and evaluating interprofessional PC teams. Thus, precise definitions, measures and instruments are needed to assess the impact of interprofessional PC teams. To the best of our knowledge, no existing review has provided a clear understanding of the terms “team functioning,” “collaboration,” “performance,” and “effectiveness” within interprofessional PC teams. By examining definitions, this review will capture the intricacies of team dynamics unique to different team-based contexts [17,43,55,56,65,66].

To help provide more clarity to the terms often used to evaluate interprofessional teams, we aim to provide a comprehensive overview of the conceptualizations, measures, and instruments associated with team functioning, collaboration, performance, and effectiveness in PC interprofessional teams. In the context of interprofessional PC teams, the aims of the proposed review are threefold:

1. To examine and analyze the conceptualizations and operationalizations of team functioning, collaboration, performance, and effectiveness
2. To identify the measures used to assess team functioning, collaboration, performance, and effectiveness
3. To investigate instruments for measurement of team functioning, collaboration, performance, and effectiveness

## Methods

### Study design

We will utilize the scoping review framework developed by Arksey and O’Malley [67], which other scholars have further refined [68,69]. This approach involves five key stages: (1) articulating the research question, (2) locating pertinent studies, (3) selecting studies for inclusion, (4) extracting and reporting data, and (5) synthesizing and summarizing the results. The forthcoming scoping review will adhere to the guidelines outlined in the Preferred Reporting Items for Systematic Reviews and Meta-analysis Protocols Extension for Scoping Reviews (PRISMA-ScR) [70] (see S1 Appendix in S1 File) and Preferred Reporting Items for Systematic Review and Meta-Analysis Protocols (PRISMA-P) checklist for systematic review protocols (see S2 Appendix in S2 File) [41] adapted using guidance by JBI for best practice reporting. This review is conducted to provide insights to inform future research on the evaluation of interprofessional PC teams.

To guide our search strategy and methodology, we present initial definitions of key terms relevant to our review. These preliminary understandings include several important concepts that are essential for examining interprofessional team-based care. Team functioning refers to the dynamic processes that facilitate collaboration and interaction among team members [71]. Effectiveness denotes the extent to which interprofessional teams deliver high-quality patient outcomes or health system outcomes [62,72]. Performance is understood as the degree to which a team achieves various goals and objectives [73]. Collaboration describes the cooperative engagement of team members who work together toward shared goals, such as by having open communication [17,55]. While these definitions provide a foundational understanding to inform our search, the primary goal of this review is to analyze how these concepts are understood and utilized in the literature. We aim to critically evaluate the various interpretations and operationalizations of these terms, recognizing that they may not be explicitly defined in all studies.

#### Articulating the research question.

The impetus for this research study came from the completion of a previous scoping review on team-based PC models [14] and through discussions with PC stakeholders. It was determined that there was a need to investigate how processes and outcomes are conceptualized in teams to inform their measurement. The following research questions emerged in the context of interprofessional primary care teams: 1) How are team functioning, performance, effectiveness, and collaboration conceptualized? 2) What are measures of team functioning, performance, effectiveness, and collaboration? 3) What instruments are used to evaluate team functioning, performance, effectiveness, and collaboration? We define measures as the specific indicators or metrics used to assess the concepts of team functioning, performance, effectiveness, and collaboration [74]. Instruments are the tools used to collect data on the measures [75].

#### Locating pertinent studies.

This review will include a systematic approach to finding peer-reviewed literature. A comprehensive search across the six academic databases, PubMed, Medline, CINHAL, Cochrane Database of Systematic Reviews, Scopus, and Web of Science, will be conducted by an information specialist. Keywords such as “team functioning,” “performance measurement,” “team collaboration, “ “team effectiveness,” “primary care,” and “primary healthcare” will be utilized to ensure the inclusion of relevant studies. The research team will utilize Boolean operators ‘AND/OR’ to develop the search strategy, and the use of ‘Truncation*’ will uncover associated terms. Our search strategy will use a combination of concepts, including ‘Surveys and Questionnaires,’ ‘Nursing Teams,’ ‘Primary Health Care,’ ‘Intersectoral Collaboration,’ ‘Clinical Decision-Making,’ and ‘Team Effectiveness,’ employing keywords and phrases such as ‘interprofessional,’ ‘interdisciplinary,’ and ‘multidisciplinary.’ Boolean operators were applied to refine the search, such as using ‘AND’ to combine relevant categories and ‘OR’ to include synonyms and related terms. Specific filters were applied to limit the search to titles, abstracts, and keywords, enhancing the precision of our literature search. The final search strategy will be developed by the research team in collaboration with the information specialist, who will translate the search to the aforementioned databases. We have conducted a test search to refine our search strategy, and the results are documented in S3 File (see S3 Appendix). This preliminary search allowed us to evaluate the effectiveness of our keywords and inclusion criteria, ensuring a comprehensive approach to identifying relevant literature for our review.

To ensure a comprehensive search, the reference lists of all identified and selected articles will undergo review by one research team member in search of additional sources. A search for grey literature will be conducted with the inclusion of conference abstracts, reports from organizations focused on PC, and website information. Conference abstracts are included as they often present preliminary findings and innovative approaches within the field of primary care, which may not yet be published in full peer-reviewed articles. They provide valuable insights into emerging trends and research that align with our review objectives. This will be done by identifying PC conferences that upload abstracts, presentations, and proceedings on their websites (e.g., American Academy of Family Physicians Annual Scientific Assembly, Canadian Family Medicine Conference, Family Medicine Forum (Canada), North American Primary Care Research Group (NAPCRG) Annual Meeting, European Conference on Interprofessional Education). A review of the first 50 pages of Google Scholar will also be done. Lastly, a review will be done of government agencies (e.g., Agency for Healthcare Research and Quality (AHRQ), National Institutes of Health (NIH), Health Canada, Canadian Institute for Health Information (CIHI).)), healthcare organizations (e.g., Institute for Healthcare Improvement (IHI), Healthcare Excellence Canada (formerly Canadian Foundation for Healthcare Improvement), American Hospital Association (AHA), The King’s Fund (UK))and non-governmental organizations (e.g., American Academy of Family Physicians or the Canadian Medical Association, the Primary Care Collaborative, World Health Organization (WHO), Health Quality Ontario) that publish reports, guidelines, and research findings on their websites. In addition to the comprehensive list of grey literature sources provided, we will enhance our search by systematically reviewing the reference lists of identified articles and relevant publications. This approach will allow us to uncover additional grey literature sources that may not be included in our initial list. Sources must pertain directly to primary care topics, interprofessional collaboration, team functioning, or related concepts that align with the focus of our research.

Once grey literature sources are identified, we will screen and evaluate them for relevance and quality using criteria similar to those of academic literature. Including grey literature in the scoping review can provide a more comprehensive understanding of the topic and ensure that all relevant evidence is considered.

#### Selecting studies for inclusion.

Articles from the searches will be de-duplicated in Covidence, which will also facilitate the screening of articles [76]. All articles will be screened against the pre-defined inclusion criteria outlined below. Two independent reviewers will review each article, both during title/abstract screening and full-text screening. Any discrepancies between reviewers will be resolved through consensus during a meeting with the Principal Investigator (lead author).

We will include all studies focusing on interprofessional PC teams from World Bank higher-income countries. All articles must be available in English or French and may include empirical studies (e.g., quantitative, qualitative, mixed methods), literature reviews, systematic reviews, scoping reviews, and meta-analyses. Only articles that include a most responsible clinician (e.g., family physician, nurse practitioner, primary care pediatricians, internists etc.) and one PC provider from a different discipline working together will be included. We define the most responsible clinician as the healthcare professional who has overall responsibility for directing and coordinating a patient’s care and treatment at a specific point in time [77–79].

Commentaries, protocols, letters to the editor, opinion pieces, case reports, editorials and articles lacking original research or analysis will be excluded as these tend to offer opinions or perspectives rather than original research or data. Non-peer-reviewed publications, such as magazine articles, newspaper articles, and blog posts, will also be excluded. Furthermore, studies focusing exclusively on specialties outside of PC settings, such as hospital-based care or specialized clinics, will be excluded. Publications not available in English and French or that do not provide explicit definitions or conceptualizations of team functioning, performance, effectiveness, and collaboration will also be excluded. Finally, articles that do not involve collaboration between the most responsible care professional and at least one other PC professional from different disciplines will be excluded (Table 1).

**Table 1:** Illustrates the inclusion and exclusion criteria in line with the PICO.

Criteria	Description	Inclusion	Exclusion
Population	Primary Care Team	Individuals providing PC services in interprofessional PC teams in World Bank higher-income countries. Individuals receiving PC services from interprofessional PC teams in World Bank higher-income countries. Organizations offering interprofessional PC through teams within World Bank higher-income countries. Team includes a most responsible clinician (e.g., family physician, nurse practitioner, primary care pediatricians, internists) and one PC provider working together	Specialty care settings outside of PC, such as hospital-based care, emergency departments, or specialized clinics (e.g., oncology, cardiology, psychiatry not integrated within primary care)
Intervention	Team-Based Primary Care	Studies that conceptualize and investigate the impact of team functioning, performance, effectiveness, and collaboration within interprofessional PC teams. Research including or examining measures or instruments utilized to evaluate team functioning, performance, effectiveness, and collaboration within interprofessional PC teams. Studies exploring various interventions designed to enhance collaboration and teamwork effectiveness within interprofessional PC teams, regardless of their specific nature.	Studies unrelated to the conceptualizing or investigation of team functioning, performance, effectiveness, and collaboration within interprofessional PC teams. Research that does not include or examine measures or instruments used to evaluate team functioning, performance, effectiveness, and collaboration within interprofessional PC teams. Studies examining interventions that do not involve collaboration between the most responsible PC provider and at least one other provider from a different professional discipline within the PC team (e.g., studies limited to solo practice or same-discipline collaboration) Studies examining interventions primarily implemented in settings other than interprofessional PC teams, such as specialized clinics or hospital-based care. Interventions lacking explicit relevance to the research questions concerning conceptualization, measurement, and evaluation of team functioning, performance, effectiveness, and collaboration within interprofessional PC teams.
Outcomes	The outcome of the included articles.	Studies that assess processes or outcomes related to team functioning, performance, effectiveness, and collaboration within interprofessional PC teams. Research investigating the impact of interventions on measures of team functioning, performance, effectiveness, and collaboration within interprofessional PC teams.	Studies unrelated to outcomes specifically related to team functioning, performance, effectiveness, and collaboration within interprofessional PC teams. Research that does not assess the impact of interventions on measures of team functioning, performance, effectiveness, and collaboration within interprofessional PC teams. Outcomes primarily focused on individual healthcare provider performance or patient outcomes without direct relevance to team functioning and collaboration within interprofessional PC teams. Studies examining outcomes primarily in settings other than interprofessional PC teams, such as specialized clinics or hospital-based care. Outcomes lacking explicit relevance to the research questions concerning conceptualization, measurement, and evaluation of team functioning, performance, effectiveness, and collaboration within interprofessional PC teams.
Study design	The study design (methodology) of included articles.	Articles containing original research or reviews with analysis on interprofessional PC teams. Studies providing empirical evidence, including quantitative, qualitative, mixed methods, literature reviews, systematic reviews, scoping reviews, and meta-analyses. Research articles that explicitly address the conceptualization, measurement, and evaluation of team functioning, performance, effectiveness, and collaboration within interprofessional PC teams.	Commentaries, protocols, letters to the editor, opinion pieces, case reports, editorials, and articles lacking original research or analysis. Non-peer-reviewed publications, such as magazine articles, newspaper articles, and blog posts. Publications not focused on interprofessional PC teams or lacking relevance to the research questions concerning team functioning, performance, effectiveness, and collaboration. Studies without empirical evidence or lacking in-depth analysis of interprofessional collaboration within PC teams. Articles that do not contribute knowledge or insights to the field of interprofessional PC teamwork.
Publication Language	The language of included articles	Published in English or French	Published in languages other than English or French
Time Period	The time period of included articles	Published from 2000 onwards.	Published before 2000

The study selection process will be documented using a PRISMA flowchart [70].

#### Data extraction and charting.

A data extraction form will be kept in Covidence [76]. We will first test the data extraction in duplicate on the first 5% of articles. Any changes will be incorporated. Once the research team devises a final data extraction form, the reviewers will extract the data independently (i.e., not in duplicate). Data extraction will include the essential details of the study (authorship, publication year, country of origin, objectives, research design, findings (impact on outcomes, barriers, and facilitators), limitation); and conceptualization of team functioning, effectiveness and performance and collaboration. We will also capture information on the practice (model, funding, governance, service requirements, panel size, characteristics of population, size of community, local shared services) and interprofessional team (most responsible provider, team members, length of employment, size, composition, scope of practice, leadership structure, co-location, provider experience).

### Quality assessment

All articles meeting the inclusion criteria will be included in the scoping review. Articles will not be excluded due to quality. However, during the data extraction phase, all included articles will undergo assessment using the Mixed Methods Appraisal Tool (MMAT) version 2018 to evaluate their methodological quality [80]. This assessment will be conducted independently by two reviewers, trained in qualitative, quantitative and mixed-methods research methods in duplicate to ensure consistency and reliability. Any discrepancies between the reviewers’ assessments will be resolved through discussion and consensus. In cases where consensus cannot be reached, a third reviewer (the Principal Investigator) will be consulted to adjudicate the discrepancies.

The MMAT is deemed suitable for this review because it is specifically designed to assess the methodological quality of various types of studies, including qualitative, quantitative, and mixed-methods research [80]. Given that the scoping review aims to include a range of study designs, such as empirical studies, literature reviews, systematic reviews, scoping reviews, and meta-analyses, it allows for a structured and systematic appraisal process of diverse study designs, enabling reviewers to assess key methodological components relevant to each type of study included in the review [81]. While the quality assessment will provide valuable insights into the state of the evidence, our synthesis of results will include all studies in our narrative report, ensuring a comprehensive overview of the current landscape in the field.

#### Summarizing and reporting.

All articles meeting the inclusion criteria will be included in our synthesis, regardless of their quality. Data analysis will occur through a systematic approach that encompasses thematic analysis [82] and narrative synthesis techniques [83]. Thematic analysis facilitates the identification of recurring patterns and themes across studies, capturing nuances in conceptualizations and measurements of team dynamics. Narrative synthesis complements this by contextualizing findings within broader frameworks, ensuring a comprehensive understanding of interprofessional team functioning. Together, these methods support the review’s goal of synthesizing fragmented literature into a coherent narrative, while preserving the complexity and variability inherent in primary care team research.

Firstly, two researchers will organize the extracted data into categories based on key themes identified for each category of extracted data. Each team member will then individually analyze a subset of the data according to predetermined thematic categories (i.e., governance models, leadership structures, funding mechanisms, etc.). Following individual analysis, the team will convene to discuss their findings and reconcile any discrepancies or divergent interpretations. Through iterative discussions and consensus-building exercises, the team will refine the thematic categories, clarify definitions, and develop a comprehensive understanding of the data. These themes may include differences in the conceptualization of team functioning, collaboration, performance, and effectiveness as they relate to differences in organizational or practice characteristics. Within each of these themes, we will aim to identify patterns, trends, and variations across different studies based on jurisdiction, outcome measurement and team composition.

Next, we will synthesize the findings into a coherent narrative that provides insights into the conceptualizations, measures, and instruments used to assess team functioning, collaboration, performance, and effectiveness in interprofessional PC teams. This narrative will highlight key findings and identify gaps in the literature to provide a comprehensive overview of the current state of knowledge in this field and contribute to the advancement of interprofessional PC teams.

Throughout the analysis process, team members will maintain clear documentation of their observations, interpretations, and decisions that occur as a group (e.g., team meeting notes and decision logs). This documentation will serve as an audit trail to ensure transparency in the analysis process [84].

#### Stakeholder consultation.

We have gathered a stakeholder consultative team that involves Canadian clinicians (e.g., physicians, nurses, physicians allied health professionals), two patients with lived experiences of primary health care, and a decision-maker involved in health care delivery and reform. As we progress through the review, stakeholders will have the opportunity to provide feedback on preliminary findings and emerging themes. This will be facilitated through follow-up consultations, where stakeholders can discuss the relevance of the results and offer suggestions for practical applications in their respective fields. This iterative feedback process will help ensure that the review remains grounded in real-world contexts and addresses the challenges faced by stakeholders.

***Current State*:** As of the time of publication, we have completed preliminary searches to refine our search strategy and have conducted pilot testing of our data extraction forms. These steps were undertaken to ensure that our methodology is robust and that we can efficiently extract relevant data once the full review commences.

## Discussion

With the increasing adoption and promotion of interprofessional PC teams across global jurisdictions, there is a need for knowledge on how concepts such as team functioning, collaboration, performance, and effectiveness are conceptualized and measured. Prior research has explored interprofessional PC teams, with studies highlighting their potential to improve patient outcomes [9,10,16–19], workforce satisfaction [11–13], and care coordination [14,15]. However, conceptual and methodological inconsistencies in evaluating team dynamics—such as interchangeable use of terms like “team functioning” and “collaboration” [49]—limit comparability and generalizability. Thus, the purpose of this scoping review is to systematically examine and analyze these concepts, along with their measures and corresponding instruments. By systematically examining the conceptualizations, measures, and measurement tools related to team functioning, collaboration, performance, and effectiveness, our review aims to provide clarity and insight into these complex constructs. Through the adoption of clear, standardized and universally accepted definitions, researchers can cultivate a common language and framework for investigating various aspects of teamwork and team functioning. This comprehensive overview will help researchers, policymakers, funders, program consultants/designers and healthcare practitioners to better understand how these terms are defined and operationalized within PC interprofessional teams, leading to improved consistency and comparability in future research and practice efforts. Additionally, by identifying and evaluating measures and tools, this review will help inform a performance framework for the evaluation of interprofessional PC teams.

The research team will submit review findings to at least one relevant academic journal specializing in PC, healthcare management, interprofessional collaboration, and health policy, such as Journal of Interprofessional Care, BMC Health Services Research, Health Affairs, The Milbank Quarterly, Health Policy and Planning, and, Journal of Primary Care & Community Health. The Principal Investigator will also present the study findings at national and international conferences, such as the American Public Health Association (APHA) Annual Meeting & Expo, the International Conference on Primary Health Care (ICPHC), North American Primary Care Research Group (NAPCRG), Association of Family Health Teams of Ontario (AFHTO) and the Canadian Association for Health Services and Policy Research (CAHSPR) Conference. Presentations will also be made to Ontario Health, Ontario College of Family Physicians, and the Ontario Medical Association Section on General & Family Practice (SGFP).

### Limitations

This review is limited in its scope. Only articles available in English and French will be included, which may exclude relevant studies published in other languages or in non-English-speaking countries. This may limit the generalizability of findings. The review is also limited to interprofessional teams in primary care settings, and findings may not generalize to hospital-based or specialty clinic contexts. Future research could explore whether similar conceptualizations and measures apply in these settings, as well as investigate emerging trends (e.g., virtual collaboration) to expand understanding of interprofessional dynamics. The focus on predefined data extraction categories for data analysis may restrict the exploration of emerging themes or unanticipated patterns, limiting the depth of understanding of team dynamics in primary care.

## Conclusion

This scoping review protocol outlines a systematic approach to analyze and synthesize existing literature on conceptualizations, measures, and instruments used to evaluate interprofessional primary care teams. By employing the Arksey and O’Malley framework [67] alongside thematic and narrative synthesis, this study will provide a comprehensive mapping of existing literature, addressing critical gaps in terminology and methodology. The findings will inform policy, practice, and future research, and potentially offer a standardized foundations for evaluating team-based primary care models.

## Supporting information

S1 File Systematic reviews and meta-analysis protocols extension for scoping reviews (PRISMA-ScR). (DOCX)

S2 File Preferred reporting items for systematic review and meta-analysis protocols (PRISMA-P) checklist for systematic review protocols. (DOCX)

S3 File Search strategy. (DOCX)
