# Supplementary material for: Evaluating interprofessional primary care teams in high-income countries: A scoping review protocol on the conceptualization and measurement of team functioning, effectiveness, performance and collaboration in primary care
Source: PLoS One. 2025 Jul 18;20(7):e0328708. doi: 10.1371/journal.pone.0328708 (PMC12273952; doi:10.1371/journal.pone.0328708)
Supplement: S3 File — (DOCX) [file pone.0328708.s003.docx]

Ovid MEDLINE: Epub Ahead of Print, In-Process & Other Non-Indexed Citations, Ovid MEDLINE® Daily and Ovid MEDLINE® <1946-Present>

1     exp "Surveys and Questionnaires"/ or "Surveys and Questionnaires".mp. or (exp Psychometrics/ or Psychometrics.mp.)      1296744

2     exp Nursing, Team/ or Nursing, Team.mp. or exp Patient Care Team/ or Patient Care Team.mp.      75164

3     (exp Primary Health Care/ or Primary Health Care.mp. or (exp Family Practice/ or Family Practice.mp.) or (exp Comprehensive Health Care/ or Comprehensive Health Care.mp.) or (exp General Practice/ or General Practice.mp.) or (exp Community Health Services/ or Community Health Services.mp.) or Pediatrician.mp. or Primary health care/ or (exp interprofessional relations/ or cooperative behavior/) or patient care team/ or (exp interprofessional relations/ or cooperative behavior/ or primary care nursing/ or physicians, primary care/) or patient care team/ or (interprofessional or interdisciplinary or multidisciplinary).tw,kf. or Primary health care/ or (interprofessional or interdisciplinary or multidisciplinary).tw,kf. or ((interprofessional or interdisciplinary or multidisciplinary) adj3 (team* or network*)).tw,kf. or ((multidisciplinary or interdisciplinary or interprofessional) adj3 ('health team*' or 'care team*' or team*)).tw,kf. or Family Practice/) and (interprofessional or interdisciplinary or multidisciplinary).tw,kf. [mp=title, book title, abstract, original title, name of substance word, subject heading word, floating sub-heading word, keyword heading word, organism supplementary concept word, protocol supplementary concept word, rare disease supplementary concept word, unique identifier, synonyms, population supplementary concept word, anatomy supplementary concept word] 193781

4     exp Intersectoral Collaboration/ or Intersectoral Collaboration.mp. or (exp Cooperative Behavior/ or Cooperative Behavior.mp.) or (exp Interdisciplinary Communication/ or Interdisciplinary Communication.mp.) or (exp Cooperative Behavior/ or Cooperative Behavior.mp.)    62512

5     exp Clinical Decision-Making/ or Clinical Decision-Making.mp. or exp "Attitude of Health Personnel"/ or "Attitude of Health Personnel".mp. or exp Group Processes/ or Group Processes.mp. or exp Group Dynamics/ or Group Dynamics.mp. [mp=title, book title, abstract, original title, name of substance word, subject heading word, floating sub-heading word, keyword heading word, organism supplementary concept word, protocol supplementary concept word, rare disease supplementary concept word, unique identifier, synonyms, population supplementary concept word, anatomy supplementary concept word] 361900

6     "team effectiveness".ti,ab,kf.      406

7     ((team or group or practice or clinic) adj3 (performance or effective or effectiveness or function*)).ti,ab,kf.   71042

8     interdisciplinary communication/ or interprofessional relations/ or interdisciplinary communication/ or (inter-professional or inter-disciplinary or multi-disciplinary).kf,tw.     82855

9     2 or 3      249505

10    4 or 5 or 6 or 7 or 8   528692

11    1 and 9 and 10    5625
